# The dynamic functions of IRF4 in B cell malignancies

**DOI:** 10.1007/s10238-022-00968-0

**Published:** 2022-12-10

**Authors:** Rossana Maffei, Stefania Fiorcari, Claudio Giacinto Atene, Silvia Martinelli, Nicolò Mesini, Flora Pilato, Ivana Lagreca, Patrizia Barozzi, Giovanni Riva, Vincenzo Nasillo, Ambra Paolini, Fabio Forghieri, Leonardo Potenza, Tommaso Trenti, Enrico Tagliafico, Mario Luppi, Roberto Marasca

**Affiliations:** 1 Department of Laboratory Medicine and Pathology, Diagnostic Hematology and Clinical Genomics, AUSL/AOU Modena, Modena, Italy; 2 Department of Medical and Surgical Sciences, Section of Hematology, University of Modena and Reggio Emilia, Modena, Italy

**Keywords:** IRF4, B cell development, Kinetic control, B cell neoplasia

## Abstract

The trajectory of B cell development goes through subsequent steps governed by complex genetic programs, strictly regulated by multiple transcription factors. Interferon regulatory factor 4 (IRF4) regulates key points from pre-B cell development and receptor editing to germinal center formation, class-switch recombination and plasma cell differentiation. The pleiotropic ability of IRF4 is mediated by its “kinetic control”, allowing different IRF4 expression levels to activate distinct genetic programs due to modulation of IRF4 DNA-binding affinity. IRF4 is implicated in B cell malignancies, acting both as tumor suppressor and as tumor oncogene in different types of precursors and mature B cell neoplasia. Here, we summarize the complexity of IRF4 functions related to different DNA-binding affinity, multiple IRF4-specific target DNA motif, and interactions with transcriptional partners. Moreover, we describe the unique role of IRF4 in acute leukemias and B cell mature neoplasia, focusing on pathogenetic implications and possible therapeutic strategies in multiple myeloma and chronic lymphocytic leukemia.

## Introduction

IRF4 is a member of interferon regulatory factor (IRF) family of transcription factors, also called Pip, LSIRF, ICSAT and MUM1. It is a 19.7-Kb gene located at the 6p25.3 locus. Members of the IRF family (IRF1 through-9) are characterized by two conserved functional domains joined by a flexible linker: an N-terminal helix-turn-helix DNA-binding domain (DBD) with a unique tryptophan pentad repeat and a C-terminal interferon activation domain (IAD) critical in mediating protein–protein interactions. The DNA-binding activity of IRF4 relays on the formation of homo- or heterodimers with multiple partners that increase the DNA affinity. Differently from other IRF proteins, IRF4 binds DNA with low affinity due to an autoinhibitory conformation and needs different partners to relieve the inhibitory mechanism and recognize the DNA sequence containing Ets-IRF composite elements (EICEs) or AP1-IRF-consensus elements [1]. However, when at high concentrations, IRF4 regulates genes containing ISRE sites, presumably by homodimerization, and this property is critical for plasma cell differentiation [2].

IRF4 is expressed in cells of immune system, including lymphocytes, dendritic cells, and macrophages, in which it can regulate several functions such as proliferation, apoptosis and differentiation. IRF4 is an essential factor that controls several stages of B cell development including pre-B cell development, receptor editing, germinal center (GC) formation, class-switch recombination (CSR), and plasma cell (PC) differentiation. Mice with germline deletion of IRF4 show a differentiation arrest at the transition from immature to mature B cells, thus lacking to generate the progeny of germinal center B cells and plasma cells. IRF4-deficient mice showed impairments in immunoglobulin production and in antibody response. In addition, cytotoxic and antitumor response by T cells were reported to be affected in mice deficient in IRF4 [3].

The heterogeneity of regulated functions is due both to alternative interactions with several cofactors including PU1, E47, IRF8 and STAT6 and to a graded expression throughout B cell development and maturation. In B cell population, IRF4 has a biphasic function acting both during early B cell development and in mature B cells during germinal center reaction after antigen engagement. IRF4 controls the sequential rearrangement of immunoglobulin loci to generate a functional B cell receptor (BCR) restraining pre-B cell proliferation and influencing pre-B cell positioning inside bone marrow niches. Furthermore, IRF4 participates to the intermingled network of signals that define the cell fate of mature B cells upon antigen engagement toward apoptosis or plasma cell differentiation throughout the regulation of germinal center formation and affinity maturation.

In this review, we summarize the recent advances in the definition of the pleiotropic functions of IRF4 during early B cell development and in mature B cells. Then, we describe the unique role of IRF4 in acute leukemias and B cell mature neoplasia, focusing on biological mechanisms and possible therapeutic strategies in multiple myeloma (MM) and chronic lymphocytic leukemia (CLL). Therapeutic nucleic acid-based approaches, including antisense oligonucleotides (ASOs), are promising strategies offering the potential to target transcription factors, like IRF4, that have proven to be intractable to alternative drug modalities.

## IRF4 controls early B cell development in redundant manner with IRF8

During the development, B cells engage the sequential rearrangement of immunoglobulin loci. The H chain locus is rearranged at the pro-B stage, while the L chain locus at the pre-B stage. After the generation of a productive heavy (H) chain, it interacts with surrogate light (L) chain Vpre-B, forming a pre-BCR on the cell surface. The pre-B cells firstly undergo a clonal expansion phase characterized by high proliferation rate, followed by a resting phase, in which cells arrest their proliferation and proceed to L chain rearrangement, thereby generating IgM + B cells. The BCR is subsequently expressed on the surface of immature B cells and autoreactive cells are culled by central tolerance mechanisms [4].

IRF4 is involved in early B cell development. IRF4 acts at the two key stages of pre-B cells, negatively regulating pre-B cell expansion and promoting L chain rearrangement and transcription, directly binding to Ig kappa (k) and lambda enhancer. IRF4 was originally discovered as the partner of the Ets transcription factor PU.1 in the immunoglobulin k light chain enhancer [5]. The IRF family member IRF8 also interacts with PU.1 and acts redundantly with IRF4 in early B cell development. Both IRF4 and IRF8 interact very weakly to IRF DNA-binding sites but are recruited to EICEs through interaction with other transcription factors related to ETs family, PU.1 and Spi.B. These heterodimeric complexes are implicated in the control of Ig L chain transcription [5–8]. In the absence of both IRF4 and IRF8, B cell development is arrested at the proliferative stage of pre-B cells, failing to down-regulate pre-BCR [9]. IRF4 and IRF8 regulate the switch between cycling pre-B cells and immature B cells by downregulating the expression of surrogate light chain genes and concomitantly promoting conventional light chain rearrangement and transcription [10]. IRF4 also collaborates with the transcription factor FOXO1 to reactivate Rag gene expression critical for recombination of IgL chain [11, 12].

IRF4 together with its partner IRF8 negatively controls pre-B cell proliferation by inducing the expression of the transcription factors IKAROS and AIOLOS. These factors down-regulate MYC while promoting the expression of the cell cycle inhibitor p27^KIP^ [13]. Moreover, IRF4 attenuates the pre-B cell expansion by limiting IL-7 receptor signaling*.* IRF4 increases the expression of the chemokine receptor CXCR4, promoting the migration of cycling pre-B cells to niches with low level of IL-7 to decrease the proliferative signal [14]. IL-7 signaling counteracts pre-B cell differentiation by directly repressing light chain rearrangements [15]. Therefore, the chemotaxis of pre-B cells to niches with low levels of IL-7 would be relevant to restrain their expansion and to initiate productive light chain rearrangements [14, 15]. IRF4-CXCR4 feedforward loop would be implicated in B cell migration into CXCL12-rich BM niches, reducing the expression of mediators of B cell proliferation MYC and STAT5, while inducing IRF4 expression and light chain rearrangement [16]. Furthermore, IRF4 has a unique role in inducing the pre-B cell marker CD25, limiting IL-7 responsiveness, and promoting migration to CXCR4 [17].

## IRF4 deficiency contributes to transformation of acute leukemias

Given its role as a key transcription factor limiting pre-B cells expansion and favoring pre-B cell differentiation, IRF4 functions as a tumor suppressor against pre-B cell transformation. IRF4 is expressed at low levels in certain myeloid and early lymphoid B cell malignancies [18–20]. However, IRF4−/− mice do not generate pre-B acute lymphoblastic leukemia (ALL), but IRF4 deficiency promotes leukemogenesis in mouse model in cooperation with oncogenes such as BCR-ABL [21] and MYC [22]. In particular, IRF4 deficiency accelerates the progression of BCR-ABL-positive B-ALL in mice, and its forced up-regulation suppresses transformation both in vitro and in vivo, negatively regulating cell cycle progression. IRF4 is up-regulated in blast cells transformed by the BCR-ABL oncogene during treatment with BCR-ABL tyrosine kinase inhibitors [21]. Accordingly, microarray analysis showed low IRF4 mRNA levels in patients with Ph^+^ B-ALL [20]. Moreover, MYC-induced leukemia was greatly accelerated in IRF4 ± deficient mice showing hyperproliferative large leukemic pre-B cells resistant to apoptosis. The deficiency of IRF4 accelerates the loss of p27^KIP^ which restrains cell cycle progression [22].

IRF4/IRF8 double deficient mice develop an aggressive chronic myelogenous leukemia-like disease at early age with expansion of granulocyte–monocyte progenitors. Then, all mice die of B-lymphoblastic leukemia/lymphoma [23]. Partial block at the transition from pre-B cells to immature B cells characterizes PU.1/IRF4-deficient mice. Of note, all PU.1/IRF4 and about 50% PU.1/IRF8 double deficient mice developed pre-B ALL with reduced expression of the tumor-suppressor genes IKAROS, Blnk and Spi-B. Restoration of IKAROS and Spi-B expression reduced leukemic cell growth [24]. Very recently, Das Gupta and colleagues described the spontaneous emergence of pre-B leukemia in IRF4−/− mice with age, showing clonal preB-I mononuclear cells infiltrating bone marrow, lung, and liver. Enlarged pre-B cell compartment is detected already in healthy IRF4−/− mice, due to unrestrained proliferation in response to IL-7, suggesting the presence of a preleukemic population vulnerable to immortalization. Due to unchecked growth of preleukemic cells and activation-induced deaminase (AID) induction, a second acquired genetic alteration may arise in some cases to promote leukemia development. The IL7-JAK-STAT signaling was found to be altered by mutations in JAK1 and JAK3 genes [25]. Furthermore, the oncomir microRNA-125b is up-regulated in several types of leukemias, including acute myeloid leukemia (AML) and B-ALL and is reported to inhibit IRF4 expression while inducing tumorigenesis in hematopoietic progenitor cells and myeloid and B cell neoplasms [26].

## IRF4 is involved in plasma cell differentiation

Upon antigen engagement, activated mature B cells generate GC where they undergo affinity maturation, isotype switching and terminal differentiation of PCs. GCs are transient follicular structures which generate inside secondary lymphoid tissue to develop a T cell-dependent B cell activation upon antigen engagement. A complex network of signaling pathways is intermingled to control key processes inside GC. IRF4 and IRF8 are factors known to control GC formation, CSR, somatic hypermutation (SHM) and plasma cell differentiation in a non-redundant manner [27]. The generation of GC B cells and the development and differentiation of plasma cells are processes orchestrated by the alternate programs of gene expression regulated the reciprocal negative feedback of BCL6 and BLIMP1. PC differentiation is mainly regulated by the zinc finger transcription factor BLIMP1 and consists of a huge expansion of endoplasmic reticulum and increased protein synthesis. Moreover, BLIMP1 reduces GC program by lowering BCL6 and AID expression and represses the expression of PAX5, leading to derepression of XBP1 which induces the transcription of many genes encoding chaperones and enzymes necessary to the correct functionality of secretory apparatus. In addition, BLIMP1 regulates the mechanism of processing of heavy chain pre-mRNA to generate a transcript encoding secreted immunoglobulins.

**Fig. 1:**
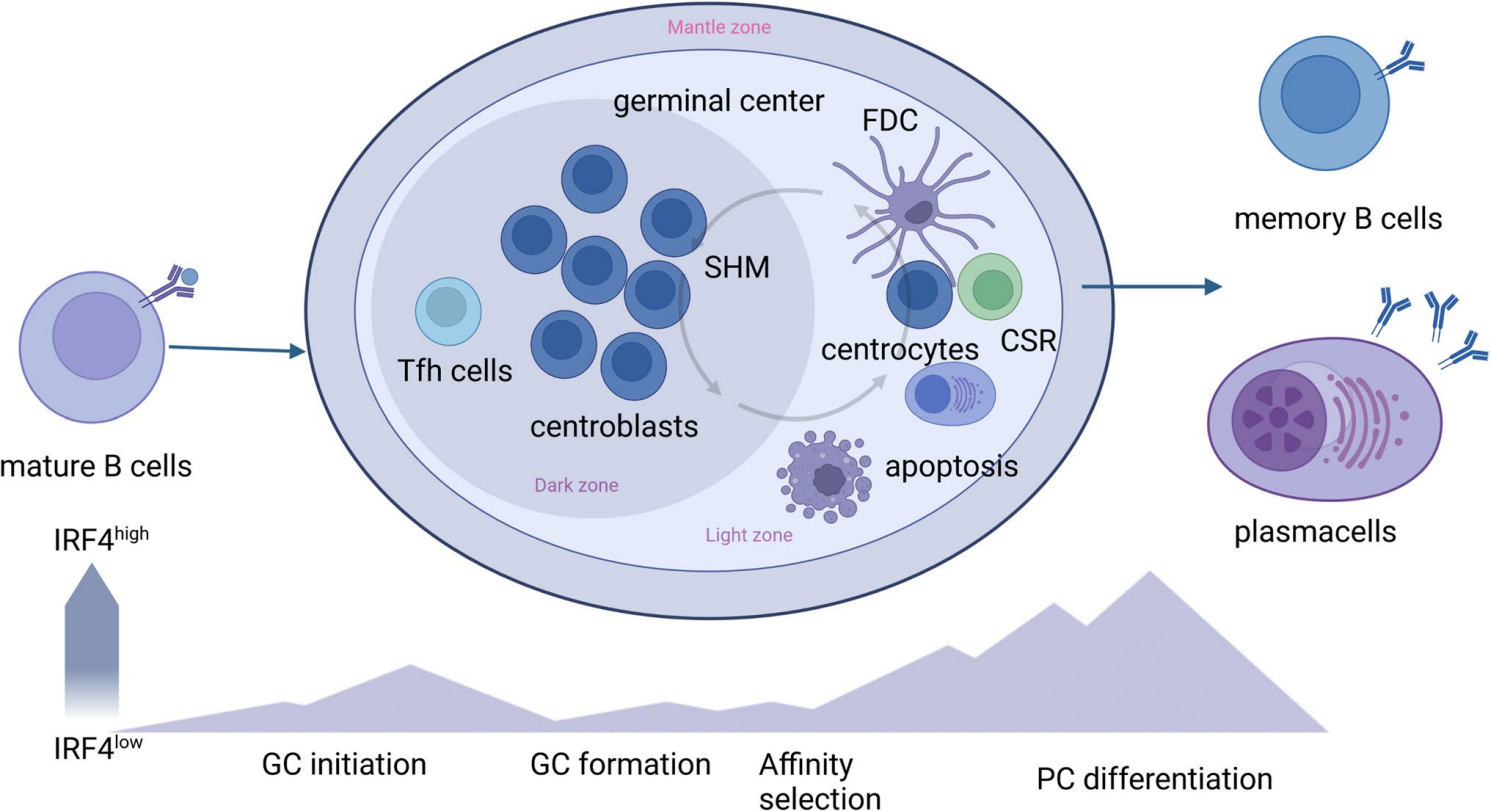
IRF4-graded expression in mature B cells during germinal center reaction. After antigen engagement, mature B cells initiate the germinal center reaction where proliferating centroblasts are regulated by high expression levels of BCL6, PAX5 and AID but low levels of IRF4. Reduced IRF4 levels also favor the localization of cycling B cells in the dark zone by regulating CXCR4 expression. Following antigen affinity maturation, IRF4 levels are progressively increased favoring class-switch recombination (CSR), mobilization of B cells to the light zone, and plasma cell (PC) differentiation. High IRF4 levels activate the PC transcriptional program by lowering BCL6 and inducing BLIMP1 and XBP1. Abbreviations: SHM, somatic hypermutation; CSR, class-switch recombination; FDC, follicular dendritic cells; PC, plasma cells; GC, germinal center; Tfh: follicular helper T cells

IRF4 is required for initiation but not maintenance of GC, by inducing BCL6 expression. Furthermore, IRF4 is required for generation of plasma cells, acting in coordinated manner with the transcriptional repressor BLIMP1, upstream of XBP1 (Fig. 1). Transgenic mice with conditional deletion of IRF4 in germinal center B cells do not form post-germinal center plasma cells. Moreover, IRF4-deficient B cells had reduced expression of AID and showed impairment in CSR [28]. In fact, IRF4−/− cells stimulated with CD40 and IL4 to induce CSR do not generate IgG1 + cells due to the low level of AID expression.

**Fig. 2:**
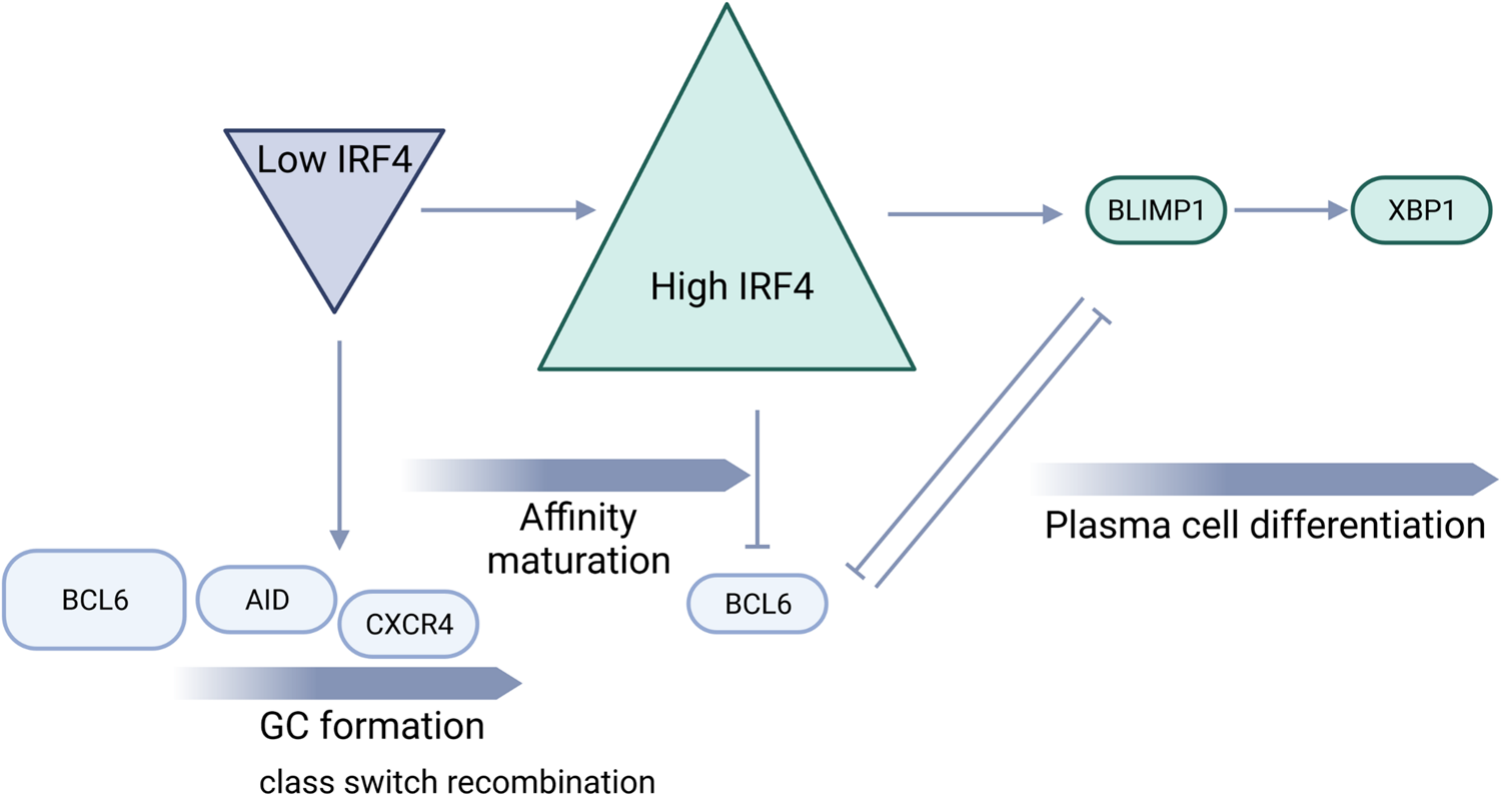
IRF4 “kinetic control model”. Fluctuation of IRF4 concentration inside germinal center controls the establishment of specific genetic programs governing germinal center reaction vs. plasma cell differentiation. Low IRF4 levels allow the maintenance of high expression of BCL6, PAX5, AID and CXCR4 allowing GC formation and class-switch recombination. Upon affinity maturation, cycling B cells increase IRF4 expression above an “on–off threshold” which conversely induces BCL6 down-regulation together with BLIMP1 over-expression promoting plasma cell differentiation. Abbreviations: GC, germinal center

However, fluctuations of IRF4 concentration in B cells underlie the generation of alternative fate, also known as “kinetic control model” (Fig. 2). According to this model, the rate of IRF4 expression upon BCR stimulation regulates the duration of AID expression, leading to CSR and SHM. Whether IRF4 accumulates in B cells above a critical threshold, it can activate Prdm1 gene (encoding BLIMP1) promoting plasma cell differentiation. Increased antigen affinity enhances BCR-mediated expression of IRF4 [29, 30]. High IRF4 concentration, allowing homodimerization, results in DNA binding at interferon sequence response motifs (ISRE) enriched in genes involved in PC differentiation.

Mechanisms of CSR and PC differentiation are strictly coordinated by the ability of IRF4 to control the expression of both Aicda and Prdm1 genes, encoding AID and BLIMP1, respectively. IRF4 is expressed in a graded manner with higher concentrations of IRF4 inducing BLIMP1 expression and transition of B cells from a GC program to that of plasma cell, whereas lower levels of IRF4 activating isotype switching/CSR and SHM by inducing AID expression [31]. Genome-wide analyses demonstrated that IRF4 regulates the entire BLIMP1-dependent plasma cell program and is involved in isotype switching process by inducing AID. Acting in a stepwise manner, IRF4 can regulate two antagonist developmental states. When B cells are stimulated by LPS- or CD40/IL4 to promote B cell activation, IRF4 expression is rapidly induced throughout several cell division, but just the appearance of an IRF4-high expressing sub-population is associated with plasma cell program.

## Increased IRF4 expression: addiction to IRF4-regulated genetic program in multiple myeloma and diffuse large B cell lymphoma

IRF4 is highly expressed in MM cells, often as a result of activating mutations or translocations and is strictly required for MM survival. IRF4 mRNA expression is an independent risk factor for poor survival, particularly in cases without 13q deletion [32]. About 20% of cases harbor the chromosomal translocation t(6;14)(p25;q32), which juxtaposes the immunoglobulin heavy chain locus to IRF4 [33, 34]. In addition, mutations in the DNA-binding domain of IRF4 gene were reported in MM cells, particularly in recurrent “hot-spots” L116R and K123R [35]. However, most MM do not have genetic lesions in the IRF4 locus but are nonetheless addicted to the aberrant genetic program regulated by IRF4 [36]. Using a loss-of-function, RNA-interference-based genetic screen, IRF4 inhibition was reported to interfere with survival of several myeloma cell lines. The IRF4-regulated network in MM cells comprises the up-regulation of over 100 genes that are quiescent in healthy plasma cells, generating an abnormal transcriptional profile more closely similar to the genetic program of antigen-stimulated B cells. The direct IRF4 targets MYC, SCD, SQLE, CCNC and CDK6 are not highly expressed in normal plasma cells but are induced in mature B cells on antigen receptor signaling activation. The pleiotropic program regulated by IRF4 in MM cells also comprises genes influencing metabolic control, membrane biogenesis, cell cycle progression, and plasmacytic differentiation.

A noteworthy target gene of IRF4 is MYC. IRF4 binds to MYC promoter region inducing its expression. A positive autoregulatory feedback loop is created when MYC up-regulates IRF4 by interacting to its intronic region. MYC expression in MM plasma cells is unusual since normal plasma cells do not express MYC due to the repression by BLIMP1. An alternative form of BLIMP1, called BLIMP1β, was reported to be over-expressed in MM cell lines. BLIMP1β is a truncated form lacking the first 101 amino-terminal residues, showing a reduced capacity to repress MYC. The expression of the truncated form of BLIMP1 can explain the inability of BLIMP1 to repress MYC in MM cells [37]. As a consequence, MYC over-expression promotes B cell activation and sustains MM survival. Furthermore, enforced expression of miR-125b-5p promotes IRF4 downregulation and impairment of its downstream effectors, reducing the growth of primary MM cells and MM cell lines [38]. Loss of IRF4 through CRISP-Cas9-mediated deletion affects MM viability and proliferation. Moreover, IRF4-regulated genes implicated in cell survival (KLF2, BCMA, MYB and MYC) were downmodulated upon IRF4 deletion, whereas pro-apoptotic factors BCL2-modifying factor (BMF) and BCL2L11 (encoding BIM) were upregulated. It implies that IRF4 affects MM apoptotic cell death by reducing the expression of pro-apoptotic factors regulating BCL2 [39]. Using a patient-derived xenograft model (PDX) of high-risk MM disease, IRF4 was reported to be highly expressed in MM progenitors and to be active in induction of several target genes involved in cell cycle progression. IRF4 down-regulation via IRF4 antisense oligonucleotide (ASO) ION251 reduced tumor formation and myeloma dissemination, eradicated myeloma progenitors and improved survival and sensitivity to myeloma drugs [40]. A phase I clinical trial of ION251 in patients with relapsed/refractory MM (NCT04398485) is ongoing.

Diffuse large B cell lymphoma (DLBCL), the most common subtype of non-Hodgkin lymphoma, is clinically and biologically heterogeneous. This heterogeneity depends on the stage of B cell development from which the disease derives (COO, cell of origin) and the activity of different biological pathways. The classification of DLBCL based on gene-expression profile related to the cell-of-origin defines 2 broad categories, the germinal center B cell (GCB)-like DLBCL and the activated B cell (ABC)-like DLBCL, with about 15% of DLBCL in the “unclassified” category [41–44]. More recently, a genetic classification based on mutations, copy-number variation and structural variants dissects DLBCL into seven genetically defined categories [45–47].

The hallmarks of ABC-DLBCL are aberrant NF-κB activation and IRF4 over-expression [48]. Similar to MM, ABC-DLBCL cells are addicted to IRF4 for survival, by activating BCR-dependent NF-κB cascade. Then, a positive-feedback loop allows the aberrant BCR signaling to sustain IRF4 over-expression in ABC-DLBCL [49]. Lenalidomide inhibits ABC-DLBCL cell proliferation, by reducing BCR-dependent NF-kB activation throughout IRF4 down-regulation. Accordingly, the knockdown of IRF4 mimics lenalidomide-mediated downregulation of NF-κB activity, whereas forced induction of IRF4 expression confers resistance to lenalidomide. Inhibition of BCR signaling with ibrutinib synergizes with lenalidomide to block IRF4 and kill ABC-DLBCL cells. In 2020, the combination of lenalidomide with the cytolytic CD19 targeting monoclonal antibody tafasitamab was approved for the treatment of relapsed/refractory DLBCL [50].

The 5th edition of WHO classification recognizes as definitive entity large B cell lymphoma with IRF4 rearrangement (LBCL-IRF4). LBCL-IRF4, despite a GCB transcriptional program, is characterized by mutations in IRF4 and NF-kB-related genes, such as CARD11, CD79B and MYD88, losses of 17p13 and gains of chromosome 7 [51]. In addition, a strong expression of IRF4 is detected in LBCL-IRF4, probably contributing to NF-kB activation. However, further studies are needed to define the potential functional effect of IRF4 in this subtype of lymphoma.

## Reduced IRF4 expression: regulating activation and immune escape in chronic lymphocytic leukemia cells

**Fig. 3:**
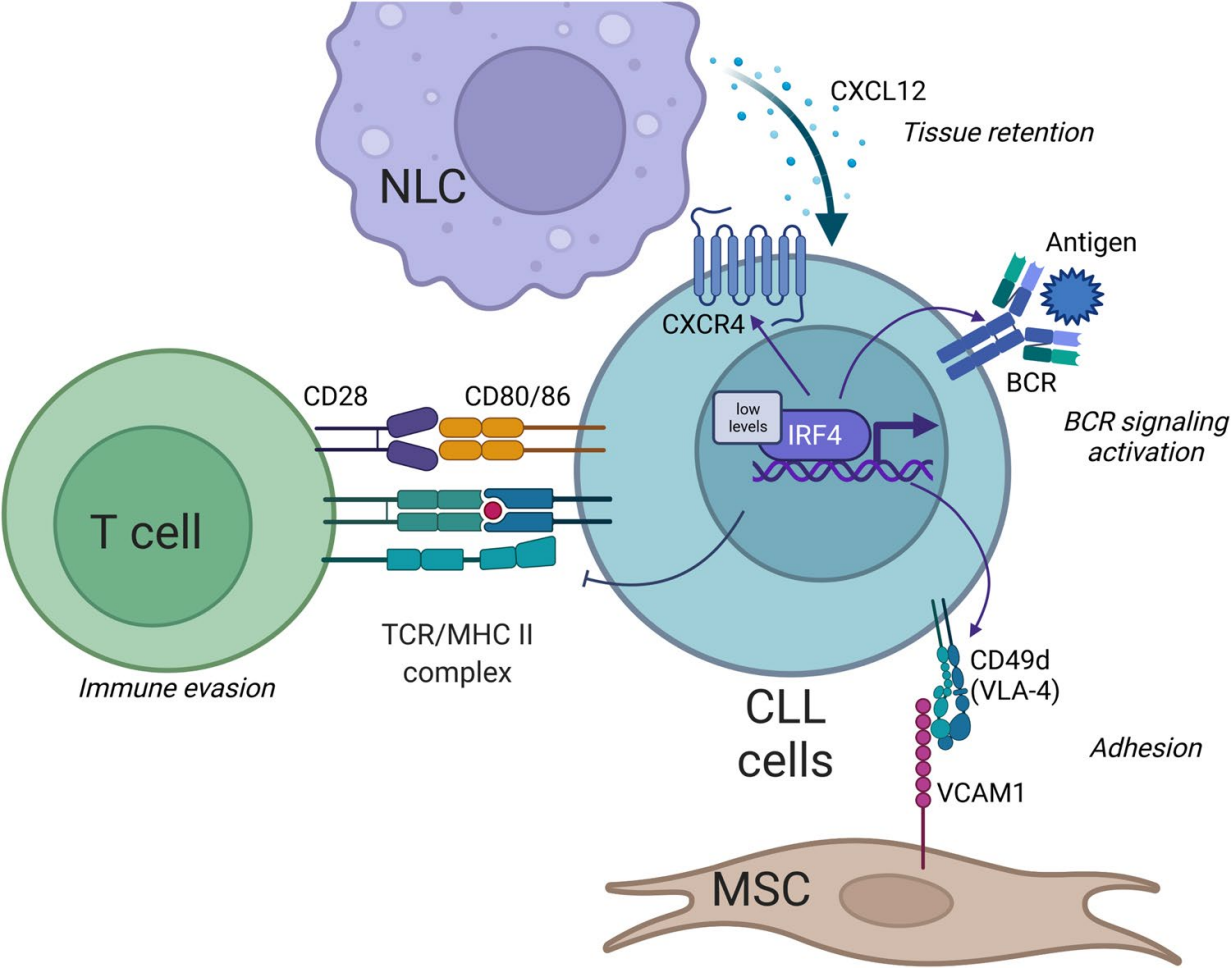
IRF4 functions in chronic lymphocytic leukemia (CLL). CLL cells show low expression of IRF4 in comparison with normal B cells. Low IRF4 level promotes the expression of molecules involved in CLL adhesion and migration such as VLA-4 and CXCR4, thus controlling leukemic cells positioning inside lymph nodes. Moreover, reduced expression of IRF4 enforces BCR signaling by regulating the expression of IKAROS and SYK. Lastly, the interaction between CLL cells and T cells is regulated by the IRF4^Low^, which decreases the expression of CD80 and CD86, thus favoring immune evasion. Abbreviations: CLL, chronic lymphocytic leukemia; NLC, nurse-like cells; TCR, T cell receptor; MHC, major histocompatibility complex; BCR, B cell receptor; MSC, mesenchymal stromal cells

Several studies suggest a possible role of IRF4 in the pathogenesis of CLL (Fig. 3). A genome-wide single-nucleotide polymorphism (SNP) association study in 517 CLL patients from the UK and 1438 British1958 Birth Cohort controls identified IRF4 as a major susceptible gene for CLL, identifying rs872071 SNP within the 3′ untranslated region (UTR) and rs9378805 SNP 10-kb centromeric to the 3′UTR of IRF4 gene as variants with the strongest association with risk to develop CLL. These findings were confirmed through two internal validation cohorts [52]. Then, rs9378805 near IRF4 and rs735665 near GRAMD1B were validated as associated with CLL risk in an independent cohort of 438 non-Hispanic Caucasian CLL [53]. Fine-scale mapping analysis identified association with CLL in 4 SNPs mapped to a 3-kb region in the 3’-UTR of the IRF4 gene [54]. Of note, reduced IRF4 expression was associated with risk alleles, suggesting a model in which it could favor CLL development by arresting transition of memory B cells into PCs [52].

A recurrent heterozygous somatic mutation in the DNA-binding domain (DBD) of IRF4, consisting of a substitution of a leucine with an arginine at the position 116 of the amino acid sequence (p.L116R, c.347T > G), was detected in 1.2–2% of CLL patients [55–58]. Patients harboring IRF4 mutation had unmutated immunoglobulin heavy chain variable gene (IGHV) status, which is associated with adverse clinical outcome in CLL [59]. Whole-genome sequencing (WGS) and whole-exome sequencing (WES) studies by next-generation sequencing (NGS) reported recurrently mutated genes in CLL patients, including the IRF4 gene with L116R variant at a frequency ranging from 0.7 to 1.6% [60–62]. Of note, Puente et al. reported IRF4 gene mutations among novel prognostic drivers in CLL, finding association with shorter time to first treatment, independently from clinical stage and immunoglobulin mutational status [63]. IRF4 L116R mutation seems to accumulate in treated CLL patients and in CLL experiencing Richter transformation (RT) [61, 64]. IRF4 L116R mutation was found in 11% of ibrutinib-relapsed patients who had experienced RT [65]. In addition, the genomic characterization of the patient-derived tumor xenograft models of Richter syndrome revealed the L116R IRF4 mutation in the mutational profile [66]. A recent study demonstrated that IRF4 L116R mutation is functionally active conferring a proliferative advantage to CLL cells [67]. The leucine 116 is positioned in the highly conserved DNA-binding domain of IRF4 gene, and its substitution with an arginine may affect IRF4 DNA-binding properties. The L116R mutation determines a more robust binding of IRF4 to all DNA targets (ISRE, EICE, AICE), suggesting a gain-of-function mechanism. Additional analyses are required to define the specific DNA‐binding properties of IRF4 L116R protein and the oncogenic role of this missense variant in CLL transformation.

IRF4 L116R mutation is rare in untreated CLL patients. In the majority of CLL, IRF4 expression is significantly downregulated as compared with healthy individuals [68]. Moreover, patients showing low IRF4 expression had significantly decreased time to first treatment (51.3 month) compared with IRF4^high^ CLL patients (79.4 months). The negative prognostic impact of decreased IRF4 expression was also validated in 2 independent CLL patient cohorts. Furthermore, low IRF4 expression, defined by immunohistochemical stains as less than 20% CD20^+^ B cells positive for MUM1/IRF4, was reported to be associated with advanced clinical stage, diffuse marrow involvement and reduced time to first treatment (TTFT) in CLL patients. High IRF4 expression is more frequent in CLL with mutated IGHV gene and better outcome [68, 69].

The maintenance and evolution of CLL clone rely on leukemic cell positioning inside “proliferation centers” and on the efficient transmission of BCR-mediated intracellular cascade. Blocking the transmission at different nodal points leads to an effective reduction of CLL survival and exiles cells from the protective tissue microenvironment. B cells deficient of IRF4 show an enrichment of genes involved in cell migration and homing, in particular of VLA-4 [70]. In CLL cells harboring trisomy 12 aberration, low levels of IRF4 mediate VLA-4 expression throughout the regulation of IKAROS [71]. Low IRF4 levels enforce BCR signaling by inducing SYK expression and promoting the accumulation of IKAROS protein, which reduces the expression of the BCR negative regulator SHIP1 [72].

A causal relationship between low levels of IRF4 and the development of CLL was also demonstrated in mouse models [73–75]. In New Zealand Black (NZB) IRF4+/− mouse model, CLL development is dramatically accelerated and IRF4+/− CLL cells showed hyper-responsiveness to BCR stimulation [74]. Shukla et al. backcrossed Vh11 mice, which have expanded B1 cell population, into IRF4-deficient mice and found that 100% of IRF4−/− Vh11 mice developed CLL within 10 months [73]. Enhanced CLL disease progression was observed in IRF4-deficient TCL1 transgenic mice, finding a severe downregulation of genes involved in T cell activation such as MHC molecules and CD80 and CD86 [68]. This study demonstrates that IRF4 is involved in regulating the CLL/T cell interaction. Lack of IRF4 in murine CLL contributes to tumor immune evasion by reducing the numbers of antigen-experienced, potentially tumor-specific T cells and is associated with a more aggressive disease.

Overall, reduced level of IRF4 seems to improve CLL homing to lymph nodal compartment, BCR activation and tumor immune evasion, but it may also potentially contribute to differentiation arrest. However, when CLL cells acquire IRF4 mutations, rarely occurring in untreated patients, a different genetic program might be activated, conferring the trajectory to a transformed phenotype. Further studies are needed to unravel the complexity of IRF4 function in CLL cells and its contribution to CLL and Richter transformation.

## Future considerations

The dynamics of IRF4 expression influence the cell fate of B cell from the early B cell development, thought the germline formation, the transition from centroblast to centrocyte, until plasma cell differentiation. The fluid behavior of IRF4 is mediated by complex mechanisms related to different DNA-binding affinity, multiple IRF4-specific target DNA motif, and complex interactions with several transcriptional partners. IRF4 is an attractive therapeutic target in B cell malignancies, particularly in MM and CLL settings. Classical strategies involving the use of immunomodulatory drugs (IMIDs) such as lenalidomide or novel approaches comprising next-generation class of IRF4 antisense oligonucleotides (ASOs), that employ constrained ethyl residues that mediate RNase H-dependent degradation of IRF4 mRNA, mediate IRF4 down-modulation, interfering with the IRF4-regulated transcriptional program and IRF4-MYC feedback loop in MM. Conversely, over-expression of IRF4 in CLL seems to interfere with survival signals mediated by BCR activation and leukemic cell homing inside “proliferation centers”, counteracting key signals of CLL progression and clonal evolution. In this setting, exploiting the inverse effect of lenalidomide on IRF4 in CLL cells or testing all-trans retinoic acid (ATRA) to increase IRF4 expression need further investigation.
